# Stormram 4: An MR Safe Robotic System for Breast Biopsy

**DOI:** 10.1007/s10439-018-2051-5

**Published:** 2018-05-21

**Authors:** Vincent Groenhuis, Françoise J. Siepel, Jeroen Veltman, Jordy K. van Zandwijk, Stefano Stramigioli

**Affiliations:** Department of Robotics and Mechatronics, University of Twente, att. Vincent Groenhuis, Room CR-3.526, Postbus 217, 7500AE Enschede, The Netherlands

**Keywords:** Pneumatics, Magnetic resonance imaging, Stepper motor, Rapid prototyping

## Abstract

Suspicious lesions in the breast that are only visible on magnetic resonance imaging (MRI) need to be biopsied under MR guidance with high accuracy and efficiency for accurate diagnosis. The aim of this study is to present a novel robotic system, the Stormram 4, and to perform preclinical tests in an MRI environment. Excluding racks and needle, its dimensions are 72 × 51 × 40 mm. The Stormram 4 is driven by two linear and two curved pneumatic stepper motors. The linear motor is capable of exerting 63 N of force at a pressure of 0.65 MPa. In an MRI environment the maximum observed stepping frequency is 30 Hz (unloaded), or 8 Hz when full force is needed. The Stormram 4’s mean positioning error is 0.73 ± 0.47 mm in free air, and 1.29 ± 0.59 mm when targeting breast phantoms in MRI. Excluding the off-the-shelf needle, the robot is inherently MR safe. The robot is able to accurately target lesions under MRI guidance, reducing tissue damage and risk of false negatives. These results are promising for clinical experiments, improving the quality of healthcare in the field of MRI-guided breast biopsies.

**Electronic supplementary material:**

The online version of this article (10.1007/s10439-018-2051-5) contains supplementary material, which is available to authorized users.

## Introduction

### Clinical Challenge

Breast cancer is one of the most frequently diagnosed cancer types in women, with an estimated 1.67 million new cases in 2012.^14^ It is essential to detect cancer at an early stage to optimize patient outcomes. Mammography is the primary imaging modality in screening programs, followed by ultrasound. Magnetic resonance imaging (MRI) is often used in women with an increased risk for breast cancer. Lesions that are not visible on mammogram or ultrasound may be detectable on MRI which has a higher sensitivity than the other two imaging modalities.^10^ Computed tomography is not routinely used in breast cancer screening due to the additional radiation dose and the limited added value besides mammography. When a suspicious lesion is found and diagnosis needs to be confirmed the radiologist may decide to acquire a tissue sample through a biopsy.

The next step in the evaluation of MRI detected suspicious lesions is targeted ultrasound imaging and, if positive, ultrasound guided biopsy. However, the radiologist is often not capable to find the detected lesions on MRI during ultrasound scanning. This makes an MRI-guided biopsy necessary. It is difficult to target the lesion precisely during this procedure due to bore accessibility constraints that require the patient to be moved in and out of the scanner multiple times. When a positioning grid is used, discretization errors up to 4 mm are introduced.^9^ In addition, aspiration and other unintentional body movements may cause breast tissue displacements. These factors may cause false negative results, or a prolonged procedure due to repositioning of the needle.^3^,^15^

An MR safe robotic system could solve the shortcomings of existing manual MRI-guided biopsy procedures. Such a system, when placed inside the scanner bore, allows biopsies to be performed with fast MRI feedback. We expect that due to the increased accuracy fewer samples will be needed, resulting in reduced tissue damage and shorter procedure times. In view of past developments on this topic the aim of the Stormram 4 project is to design and characterize a novel MR safe robotic system for breast biopsy with significant improvements over earlier designs, especially in terms of accuracy and workspace.

### MR Classification

The ASTM F 2503 standard defines three possible classifications for MRI devices: MR safe, MR conditional and MR unsafe. The MR safe classification is assigned to devices that exclusively consist of non-metallic, non-magnetic and non-conductive materials, and can be assigned based on a scientific rationale (i.e., material composition) rather than test data. In contrast, the MR conditional classification indicates that the device is only safe when used under certain tested conditions. Finally, the MR unsafe classification indicates that the device is known to pose hazards in all MRI environments. This new standard is designed to avoid confusion and errors originating from the older terminology (MR compatible/MR safe), which are thus to be avoided in new research.

### State of the Art

Several MRI robots have been described in literature. The use of conventional electromagnetic motors are ruled out due to interference with the magnetic field of the MRI scanner. Various alternative actuation methods have been investigated: hydraulics, piezo motors, ultrasonic motors, cable transmissions, MR-driven ferromagnetics, flexible fluidic actuators, air turbines, direct-acting pneumatic cylinders and pneumatic stepper motors. Actuation by pneumatic stepper motors offers several advantages over the other categories: pneumatic stepper motors are inherently MR safe, tolerant for small air leakages, clean in medical applications, can be controlled with conventional pneumatic manifolds and allow for feed-forward control methods.

**Figure 1:**
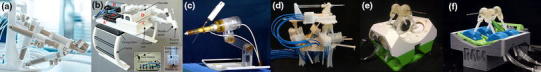
State-of-art pneumatic robots: (a) transrectal prostate biopsy robot by Bosboom And Colleagues,^2^ (b) liver tumor ablation robot by Franco *et al*.,^4^ (c) endorectal prostate biopsy robot by Stoianovici *et al*.,^12^ (d) Stormram 1 by Groenhuis and Stramigioli,^5^ (e) Stormram 2 by Groenhuis *et al*.,^1^,^7^ (f) Stormram 3 by Groenhuis *et al*.^8^

Figure 1a–1f shows six pneumatic MRI robots found in literature. (a) Bosboom *et al*. developed and tested an MR safe, remote controlled parallel manipulator for transrectal biopsy guidance.^2^ It is driven by five stepper motors. Each motor contains a rod with a helical hole pattern on which four single-acting cylinders act alternatingly, resulting in a screw movement of the rod. The robot requires a median manipulation time of approximately 6 min to move the needle guide to the commanded position, which makes it relatively slow. (b) Franco *et al*. described a needle-guiding robot for laser ablation of liver tumors.^4^ The relatively large robot is operated by four direct-acting pneumatic cylinders for which a special control scheme was developed to drive the piston accurately to the target position. Position feedback is acquired using electronic optical encoders, making it relatively complex and not MR safe. Its reported mean in-plane targeting error is 2.9 mm. (c) Stoianovici *et al*. developed an MR safe robot for endorectal prostate biopsy, driven by three pneumatic rotational stepper motors called PneuStep.^11^,^12^ It uses fiber optic quadrature encoders for position measurements. The reported needle targeting accuracy is 0.37 mm in bench test and 2.09 mm in MRI. Being specifically designed for prostate biopsies the actuated workspace of this type of robot is too limited for breast biopsy purposes.

The authors of this paper developed three earlier versions of the Stormram breast biopsy robot. Figure 1(d) shows the Stormram 1 which is driven by seven pneumatic linear stepper motors of which six form a Stewart platform and the seventh one inserts the needle longitudinally.^5^ While this robot is able to demonstrate the proof-of-principle of pneumatic stepper actuation, the whole robot is too large to fit alongside a patient in the MRI scanner. Figure 1(e) shows the Stormram 2, which is driven by smaller stepper motors integrated inside ball joints, resulting in a more compact robot. Measurements have shown that it is able to target lesions in a phantom breast with a relatively poor accuracy of 6.0 ± 2.0 mm,^1^,^7^ mainly due to clearances in the joints. Figure 1(f) shows the Stormram 3 with a similar size and kinematic design as its predecessor. While the accuracy was improved to 2 mm and a stronger needle insertion actuator delivering up to 70 N of force was installed,^8^ the parallel kinematic structure resulted in a complex control structure and suboptimal workspace.

### Approach

**Figure 2:**
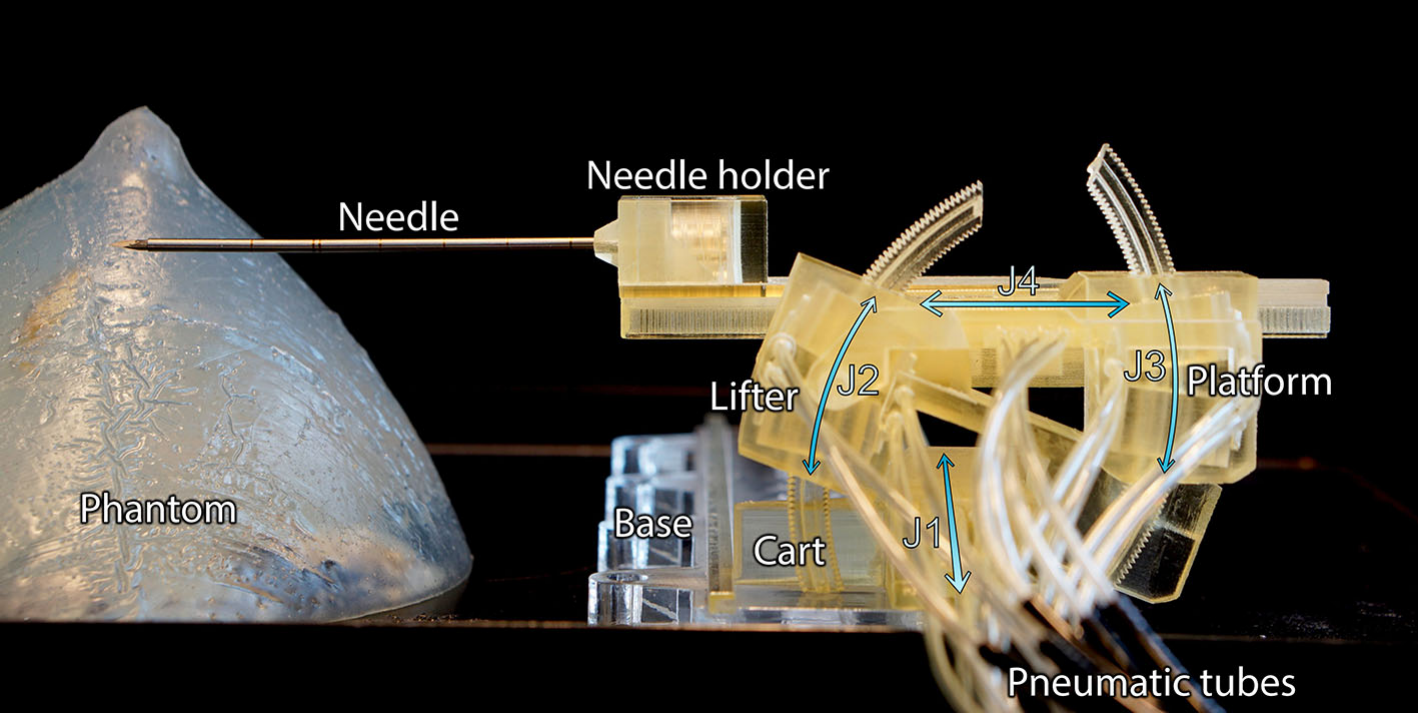
Stormram 4, with labeled parts and joints J1–J4.

This paper describes the design and evaluation of the Stormram 4, shown in Fig. 2. It was developed to address the shortcomings of the state-of-art MRI robots, specifically in terms of size, complexity, accuracy and workspace.^6^ The approach is to use a serial kinematic chain driven by a combination of linear and curved pneumatic stepper motors.

The use of a serial kinematic chain instead of a parallel one results in an enlarged workspace. High accuracy is achieved by using backlash-free joints in combination with a linear step size of 0.25 mm, and an angular step size of 0.25° (corresponding with 0.44 mm displacement at a radius of 100 mm). Good stiffness is achieved by making the robot compact and by driving revolute joints at a radius of 50 mm from its axis. Flexible, thin (2 mm) pneumatic tubes are used in the proximity of the robot to allow unconstrained movement of the different degrees of freedom. Besides bench tests, extensive MRI tests are reported. For operator-independent, precise validation of the needle placement an automated needle detection algorithm was developed.

## Materials and Methods

### Kinematic Design

The Stormram 4 is a serial kinematic manipulator with four degrees of freedom, each driven by a pneumatic stepper motor. In its home position the robot (excluding needle and racks) measures 72 × 51 × 40 mm.

The different parts of the robot are labeled in Fig. 2. The base is fixed and consists of a linear rail over which the cart can slide back and forth over a distance of 160 mm, this joint J1 is driven by a linear stepper motor. The base and lifter are linked by a revolute joint J2 which is driven by a curved stepper motor with a range of 47°. The lifter and platform are linked by another revolute joint J3 which is also driven by a curved stepper motor with a range of 38°. These two revolute joints combined allows the platform to move up and down and tilt vertically (but not horizontally). Finally, a linear stepper motor in the platform drives the needle holder longitudinally over a distance of 80 mm, forming joint J4.

### Pneumatic Stepper Motors

Two different stepper motors have been developed for the Stormram 4. The T-26 linear motor actuates a prismatic joint while the C-30 curved motor is designed to drive a revolute joint. Both motors are constructed and assembled similarly; the main difference is the radius of curvature for the rack which in turn influences the geometries of the housing and pistons.

**Figure 3:**
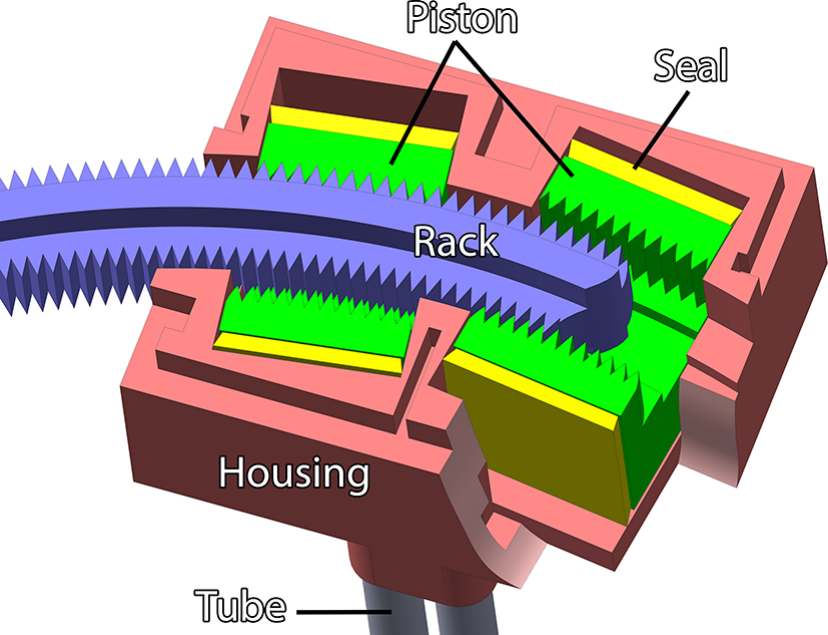
Cutaway view of the C-30 curved stepper motor, which consists of the housing, two pistons, four seals, four pneumatic tubes and the curved rack.

Figure 3 shows the internals of the C-30 curved motor. Each of the two pistons (green) is operated by delivering pressurized air into either end of the cylinder, pushing the piston up and down. Silicone seals prevent leakage of air along the pistons. A piston has two jaws (series of teeth) on the inside that engage on the rack by means of a wedge mechanism, pushing the rack to the left or right in small steps. As the rack is curved with a radius of 50 mm there exists a well-defined rotation axis which can be supported by a hinge joint, significantly increasing stiffness of the mechanism.

### Production

**Figure 4:**
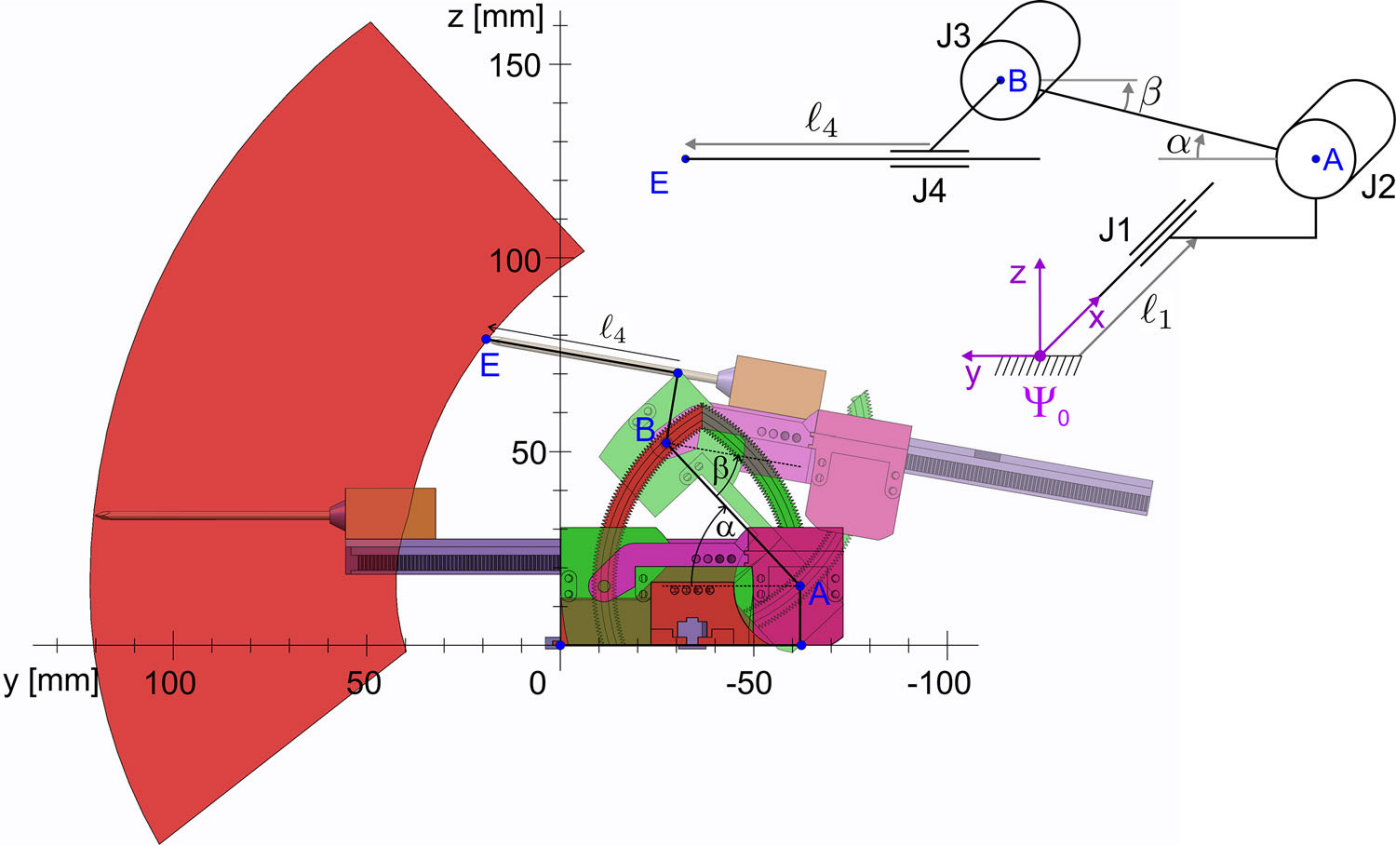
Workspace (red region), and two extreme poses of the Stormram 4. Top-right: kinematic configuration.

The majority of the robot parts were printed on an Objet Eden260 (Stratasys Ltd., Eden Prairie, MN, USA) in FullCure720 material and assembled together by gluing. The seals were laser-cut from 0.5 mm silicone rubber. Acrylic rods with a thickness of 3 mm were used in the two hinge joints between the cart, lifter and platform components located at points A and B in Fig. 4. These rods were lubricated with silicone grease to allow rotational motion with sufficiently low friction and without any measurable play. Polyurethane tubes deliver pressurized air from the valve manifold to the robot. Silicone grease serves as lubricant for the moving parts.

In this study the robot was equipped with an MR conditional Bard 14G 100 mm titanium needle (Bard, Inc., Murray Hill, NJ, USA). The diamond-shaped symmetric tip and needle rigidity resist bending due to needle–tissue interactions so that the needle can be assumed straight in all experiments. As the needle can be swapped with another one, e.g., to meet the conditions of a specific MRI scanner, the chosen needle is not considered to be an integral part of the robot. Based on the full material composition of the robot (excluding needle) the robot itself can be classified as MR safe.

The robot base consists of a laser-cut acrylic plate on which a linear rack and a guide rail are positioned. The base itself is attached to a table printed in polylactic acid in which 10 fish oil capsules are embedded as fiducials for registration and alignment purposes.

Several breast phantoms were manufactured by pouring a hot mixture of PVC and plasticizer into a 3D printed mould generated from a mathematically described breast shape.^1^,^8^ In each phantom 4–10 lesions were added (size range 5–20 mm) to the volume during the cool-off process. During this process the phantom solidifies to an elastic mass with randomly distributed lesions. For the lesions, either fish oil capsules or pieces of stiff PVC were used, both of which are relatively stiff and well distinguishable on MRI in certain sequences.

### Pneumatic Controller

**Figure 5:**
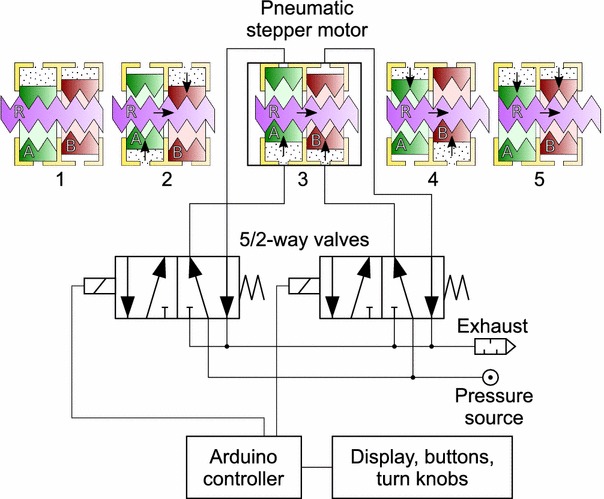
Pneumatic control schematic and five consecutive states of a single stepper motor.

**Figure 6:**
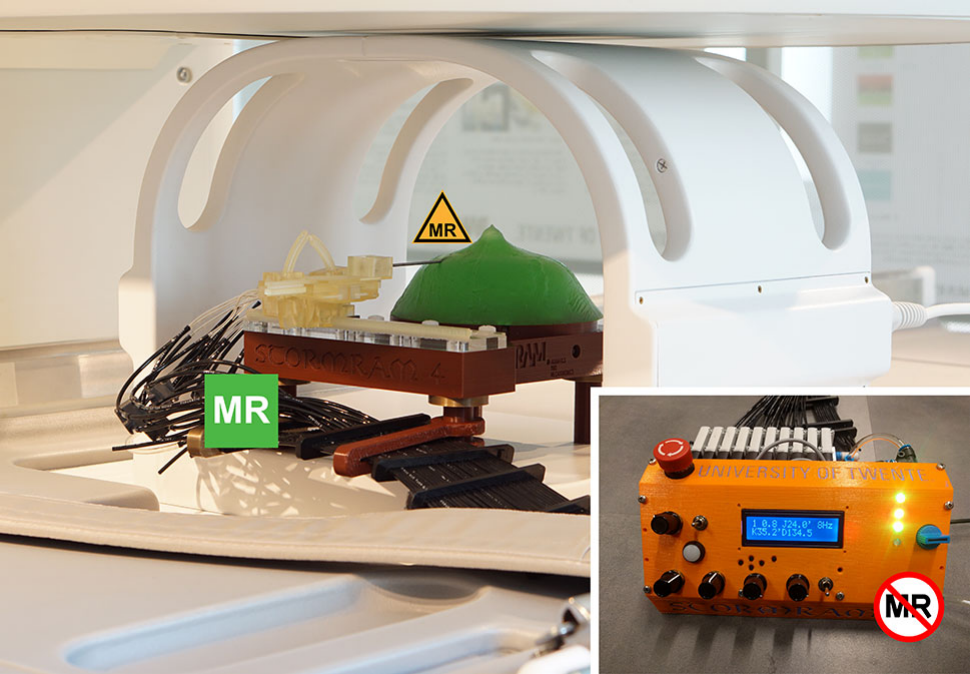
Experimental setup for MRI tests with an MR safe Stormram 4 piercing a breast phantom using an MR conditional needle. Bottom-right: MR unsafe pneumatic controller with user interface.

The robot is controlled by a pneumatic valve manifold with user interface. Figure 5 shows a schematic of the controller including one pair of valves for driving one stepper motor. A photo of the actual controller can be seen in Fig. 6 (bottom-right). Each of the four stepper motors is independently operated by a pair of 5/2-way valves of type Festo MHA2-MS1H-5/2-2 (Festo AG and Co. KG, Esslingen, Germany) connected with 5 m long pneumatic tubes. This allows the MR unsafe controller to be placed outside the Faraday cage of the MRI to eliminate the possibility of RF interference and safety issues.

The pneumatic connection scheme and operating sequence of a single stepper motor is shown in Fig. 5. Two pistons (green and red) act on a rack (purple). The five states, numbered 1–5, show the consecutive positions of the pistons and rack when it is operated from left to right. Exactly one piston can be fully engaged on the rack. This is always the piston that moved formerly and independent of the direction of load. The piston that moved most recently acts as a wedge between the rack and the cylinder housing, effectively eliminating any backlash in the motor by design. Reversal of direction results in a small hysteresis effect which could be exploited to achieve some form of sub-stepping. Alternatively, this effect could be avoided by choosing one particular direction of approach.

The sequence of valve states is controlled by an Arduino controller which maintains a memory of the current motor position for each of the four joints: (*L*_1_, *α*, *β*, *L*_4_). *L*_1_ is the displacement of joint J1 in mm. *α* is the angular displacement of joint J2 in degrees. *β* is the angular displacement of joint J3 in degrees. *L*_4_ is the linear displacement of joint J4 in mm.

When one motor is commanded to move to a new position its valves are operated for the required number of steps at a given stepping frequency. As no position sensors are used, the control strategy is purely feed-forward. Correctness of the actual position is guaranteed when no steps are skipped since the last calibration, which can be assured by providing sufficient force or torque to perform the steps, exceeding the maximum loads on each actuator by a sufficiently large safety margin. Additionally, position feedback by MRI is possible.

The maximum stepping frequency is restricted by the length and diameter of the tubes, the cylinder stroke volume, the valve’s airflow and switching speed. Up to a certain threshold frequency the performance of the motor is approximately constant; above the threshold frequency the force drops gradually until it does not move anymore at all.

### Kinematics and Workspace

Figure 4 shows the workspace and two different configurations of the Stormram 4 projected on the *Y*–*Z* plane. Using the two revolute joints and the linear needle insertion joint, the needle tip can reach targets within the red-colored region. The linear joint in the base allows movement of the robot in the *X* direction, resulting in a total workspace volume of 2.2 L. If necessary, this volume could be increased further by elongation of the linear racks or the needle.

Due to the serial kinematic chain, derivation of forward kinematics is straightforward. From angles *α* and *β*, length *L*_4_ and knowledge of the robot geometry, the *Y*- and *Z*-coordinates of the needle tip E can be calculated directly. The *X*-coordinate follows directly from the displacement *L*_1_ of the linear joint in the base.

Inverse kinematics involves derivation of the four joint coordinates (*L*_1_, *α*, *β*, *L*_4_), given the target (*X*, *Y*, *Z*) coordinates of the needle tip E. The displacement *L*_1_ of the linear joint in the base follows directly from the *X*-coordinate, leaving us with derivation of *α*, *β* and *L*_4_ from a given point E in Fig. 4. It can be noted that there are multiple solutions in general, as there are three variables and only two constraints. The needle insertion angle (*α* − *β*) can be chosen as the free parameter, upon the radiologist’ discretion to, e.g., circumvent delicate structures in the breast. The values of *α*, *β* and *L*_4_ can then be found by solving the resulting set of equations. Existence of a unique solution depends on the feasibility of the different constraints—in particular the range of motions for the different joints. To help the radiologist or operator in choosing a suitable needle insertion angle its minimum and maximum values for a given position of *E* can be calculated and displayed on an interface from which a value can be picked somewhere in between.

Stepper motors can only reach a series of discrete states. The step size for linear motors is 0.25 mm, while for rotational motors it is 0.25°. When the nearest reachable joint position is used, the resulting discretization error in *X*-direction is up to 0.13 mm, while for *Y*- and *Z*-directions the error is up to 0.39 mm with a fully extended needle.

### Segmentation and Registration

**Figure 7:**
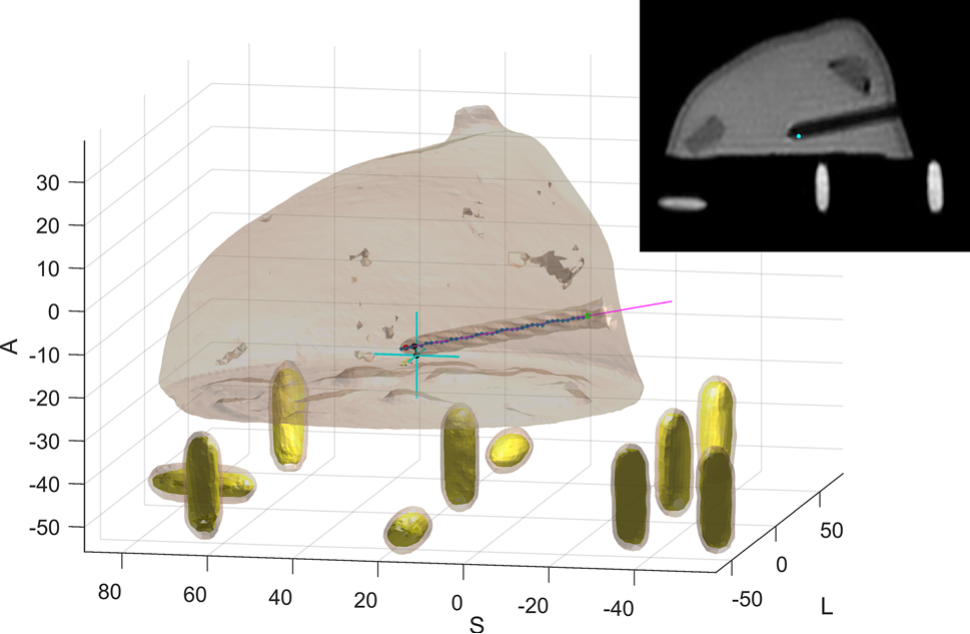
Segmentation of phantom with registration fiducials (yellow), target site (cyan), reconstructed needle segments (blue dots), best linear fit of needle centreline (magenta) and targeting error (black). Top-right: sagittal MRI slice with target site (cyan).

The left-posterior–superior coordinate system is used in DICOM images of MRI scans, with the origin being at the magnet’s isocenter. In contrast, the robot uses the *XYZ* coordinate system with the origin at a different site and the *XY* plane parallel to the MRI’s coronal plane. The associated coordinate transformation is defined by the locations of 10 fiducials, shown in Fig. 7. Seven fiducials are upright, while three lay flat.

An automatic fiducial registration method is used to save time in the procedure. First, the 10 fiducials are extracted based on the total volumes of the connected components in the geometrically corrected and binarized scan. From its sizes in posterior–anterior direction the three flat fiducials are identified and its mean position calculated from which the posterior–anterior component of the robot coordinate frame in MRI is defined. The remaining seven fiducials are registered based on intra-marker distances and, from the locations of its centroids, a best fit rigid 2D transformation in the coronal plane is constructed. Combined with the posterior component the 3D coordinate transformation is now fully defined. The mean registration error was measured to be 0.2 mm—one order of magnitude smaller than the acquisition resolution of the MRI scans.

To evaluate the accuracy of the Stormram 4 in an MRI environment the needle must be localized on the confirmation scan. In order to utilize as much voxel information as possible to enable sub-pixel localization accuracy and to save operator time this is also performed automatically. This algorithm is based on finding connected dark voxels in the binarized image. From these voxels a tree graph is constructed based on the shortest distance from the image border. After some further processing involving traversing this tree graph it is possible to recognize the needle based on its shape involving with relatively constant cross-sectional area.

### Experiments

Four experiments were conducted to characterize the force, speed and accuracy of the Stormram 4.

#### Stepper Motor Force

**Figure 8:**
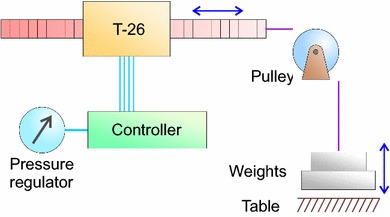
Force measurement setup. The T-26 stepper motor lifts weights of known mass, while the system pressure is adjusted using the regulator.

The maximum force of the T-26 stepper motor at low stepping frequency (< 1 Hz) depends on the operating pressure. The relationship was studied by lifting a series of weights with known mass and finding the minimum required pressure such that it is just able to lift the weight. The schematic setup is shown in Fig. 8. A linear fit is derived from the resulting pressure–force graph.

#### Maximum Stepping Frequency

The use of 5 m long tubes limits the stepping frequencies of the four motors. The maximum stepper motor frequencies for which each motor is just able to drive its joint without missing steps is recorded at a pressure of 0.25 MPa. For comparison, both short (0.5 m) and long (5 m) tube lengths were used in the test.

Next, the maximum frequency of a motor under load with 5 m long tubes was derived by pulling a weight with the T-26 motor at increasing stepping frequency until a drop in the delivered force is observed.

#### Needle Tip Accuracy in Free Air

The accuracy and precision of the needle tip in free air were evaluated by piercing 30 targets drawn as crosshairs on a vertically positioned board. For each target, the error is defined as the distance from its center to the pierced hole.

#### MRI Accuracy Tests

MRI accuracy tests were conducted on breast phantoms. Thirty different sites were identified and targeted by the needle. The coordinates of each site are given as target for the robot. The error, defined as the offset between original site location and reconstructed needle position in the robot coordinate frame, was measured for each site.

Breast deformations were not taken into account in this test. The Stormram 4 does not have a system to immobilize the breast, so the phantom on the table was allowed to deform freely resulting from needle–tissue interactions.

Figure 6 shows the experimental setup, with the Stormram 4 and phantom positioned inside a 0.25 T (G-Scan, Esaote SpA, Genoa, Italy) scanner. Taking into consideration the space requirements of the setup an abdominal coil with maximum internal height, width and length of 270, 405 and 280 mm was used. The controller was placed outside the Faraday cage of the MRI scanner connected to the robot by a total of 30 tubes with a length of 5 m. Of these 30 tubes, sixteen are used for actuating the four stepper motors while the remaining ones are reserved for future use such as the firing system of a biopsy gun.

The MRI scanner was geometrically calibrated using a custom 3D grid pattern before conducting the needle insertion experiments. From the measured pattern a fifth order correction function was defined to map observed (deformed) MRI coordinates to world coordinates. With this geometric correction, the registration error is reduced from up to 2 mm to an average of 0.2 mm within a cube-shaped volume with dimensions 180 × 180 × 180 mm, centered at the magnet’s isocenter. The correction function was applied to all observed coordinates in the tests.

A 3D balanced steady-state free precession (bSSFP) sequence was used as the scanning protocol with parameters TR = 10 ms, TE = 5 ms and FA = 60°. The scanning direction was the sagittal plane with a field-of-view of 240 × 240 mm and acquisition matrix of 160 × 160 voxels, resulting in an acquisition resolution of 1.5 × 1.5 mm in each slice. The acquired slice thickness was 2.0 mm and the (isotropic) reconstructed resolution was 0.94 mm in all directions. This scanning protocol is optimized for PVC lesions, as it shows good contrast between different tissue types in combination with a low signal-to-noise ratio.

##### Testing Procedure

First, a pre-operative planning scan was performed. The resulting scan was segmented and the robot coordinate frame was defined. Next, a series of sites within the phantom were chosen. For each site, first the robot joint configuration vector was calculated using inverse kinematics. The free parameter representing the insertion approach angle was chosen as the midpoint of the range of possible values. The robot was operated to penetrate the needle in the phantom towards the selected site by manually rotating four turn knobs of the controller. First, the J1, J2 and J3 joint coordinates were adjusted to the pre-calculated position to align the needle with the target lesion. Next, joint J4 was adjusted to insert the needle longitudinally.

After reaching the target a confirmation scan was acquired. During this scan the joint configuration vector for the next target site was also calculated and, during reconstruction of the confirmation scan, the robot was already operated towards that next target in order to streamline actions as much as possible.

The confirmation scans were analyzed using an automated script to determine the needle position and angles from which the error distances were calculated. Whenever the needle detection algorithm was unable to correctly identify the needle or the human operator observed a discrepancy between reconstructed needle and MRI scan data, the needle was reconstructed manually using the software package 3D Slicer, version 4.7.

Although the Stormram 4 was equipped with a standard needle during the tests, it could be equipped with a biopsy gun when an appropriate biopsy needle is available. Samples taken by a biopsy gun are generally long and thin, typically 15 mm in length. This implies that in targeting small lesions accurate control of the needle depth is not critical. Hence, the shortest distance of the target site to the reconstructed needle, i.e., the normal distance error, is considered as the standard error measure.^13^

## Results

### Stepper Motor Force

**Figure 9:**
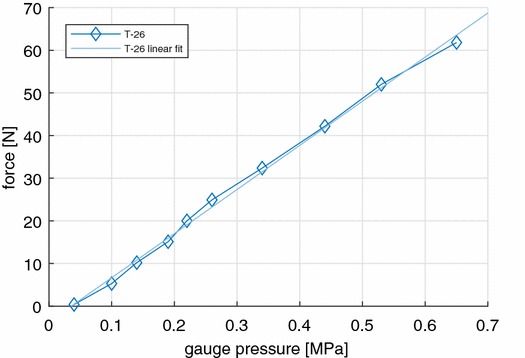
T-26 force vs. pressure.

Ten data points were collected and plotted in Fig. 9. The following linear relation was found between *F*, the measured force in Newton, and *P*, the supply pressure in Pascal:

$$F = 1.04 \cdot 10^{ - 4} P - 3.7.$$

The highest recorded force was 63 N at a pressure of 0.65 MPa. At normal operating pressure of 0.25 MPa the force is 22 N.

### Maximum Operating Frequency

The absolute maximum stepper motor frequencies of the four joints are listed in Table 1. For both short and long tubes joint J4 was found to be the limiting factor. In the case of 5 m long tubes the maximum stepping frequency was found to be approximately 30 Hz. When loaded the maximum frequency was measured to be approximately 8 Hz.

**Table 1:** Maximum stepping frequencies of the four joints of the Stormram 4 when operating in free air, using two different tube lengths.

Joints	Frequency at 0.5 m (Hz)	Frequency at 5 m (Hz)
J1	240	65
J2	240	50
J3	190	50
J4	160	30

### Needle Tip Accuracy in Free Air

Table 2 lists the observed targeting error when positioning the needle tip in free air as well as its decomposition in *X* and *Y* directions as measured by the positions of a series of 30 punctures in a vertically positioned sheet of paper with 30 marked targets. The resulting 2D error is 0.73 mm. It can be observed that accuracy in *Z*-direction is approximately four times better than in *X*-direction. Closer examination of the error data revealed that needle tip movements in *Z*-direction are slightly skewed, i.e., these movements result in slight horizontal displacements as well.

**Table 2:** Targeting error in free air given as 2D distance, and its horizontal (*X*) and vertical (*Z*) components.

2D error	0.73 ± 0.47 mm (range 0 to 1.71)
*X* error	0.46 ± 0.70 mm (range − 0.63 to 1.71)
*Z* error	0.10 ± 0.21 mm (range − 0.33 to 0.58)

### MRI Accuracy Tests

A total of 30 locations in three different phantoms were selected and subsequently targeted.

Table 3 lists the error measures and other statistics for the measurement series. The full measurement data is available as a supplementary file.

**Table 3:** Statistics summarizing the measurement results involving targeting 30 sites under MRI guidance.

Number of sites targeted	30
Targeting interval (last 10 sites)	6:38 min
Needle segmentation method	24 sites automatic, 6 sites manual
Insertion depth	25.0 ± 11.5 mm (range 8.3 to 51.3)
Apparent needle diameter	5.7 ± 0.8 mm (range 4.4 to 7.3)
Normal distance error	1.29 ± 0.59 mm (range 0.43 to 2.63)
3D distance error	1.87 ± 0.80 mm (range 0.69 to 3.57)
*X* error	− 0.37 ± 0.87 mm (range − 2.11 to 1.43)
*Z* error	0.07 ± 1.07 mm (range − 1.54 to 2.22)
Depth error	0.73 ± 1.27 mm (range − 2.67 to 3.27)
Azimuth angle error	− 0.25 ± 1.16° (range − 2.78 to 2.93)
Elevation angle error	0.44 ± 1.56° (range − 2.58 to 5.50)

In the last 10 needle insertions the interval time for successive site targets was recorded and found to be 6:38 min—equal to the total time required for one MRI scan (including pre-scan calibration and image reconstruction). This scanning time was found to be the limiting factor in the needle insertion procedure as all other actions could be performed during the different phases of the scan.

The 30 confirmation scans were segmented and analyzed using an automated script. In six cases, the script did not correctly recognize the needle location due to insufficient penetration depth and/or due to the presence of air pockets near the targeting site. In these cases the needle location was determined manually by visual inspection of the confirmation scan using 3D Slicer software.

Figure 7 shows a rendering of the confirmation scan for target site 14. The blue crosshair represents the commanded target and the needle is visible as a hole in the phantom with an apparent diameter of 5.3 mm. The blue dots represent segments of the reconstructed needle. The magenta line represents a best linear fit of the needle centerline. For this particular target the normal error was measured to be 2.38 mm.

Table 3 lists the error statistics in targeting all 30 sites. The average targeting error (shortest distance to needle) was found to be 1.29 ± 0.59 mm (range 0.43 to 2.63). When insertion depth is also considered the resulting 3D error is 1.87 ± 0.80 mm (range 0.69 to 3.57).

The error measurements for the individual needle location components (*X*, *Z*, depth, azimuth and elevation) reveal that the errors in lateral (*X* and *Z*) directions are comparable. However, there is a significant bias present in insertion depth of 0.73 mm and also in elevation angle (0.44°). The deviations in the components are about three to six times the nominal step size of the linear and rotational joints.

## Discussion

### Comparison with State-of-Art Robots

Compared with earlier robots in the Stormram line the newly developed iteration is a significant improvement in terms of workspace, accuracy, size and complexity. It is also smaller and faster than other state-of-art robots described in this paper. In terms of accuracy a fair comparison to those robots is difficult due to differences in stiffness of the targeting organ (prostate or liver) compared to that of the breast. Also, the resolution and calibration quality of the MRI scanner significantly affects the results. Comparing the Stormram 4 with the prostate robot by Stoianovici *et al*. shown in Fig. 1(c) which has a reported accuracy of 0.37 mm in bench test and 2.09 mm in MRI, the Stormram 4 obtained slightly better results in MRI.

### Kinematics

The choice to use a serial kinematic chain instead of a parallel manipulator has shown to have positive effects on size, complexity, accuracy and workspace. It has only four joints, which are all directly actuated. The revolute joints incorporate high stiffness thanks to the actuation method by curved stepper motors.

### Stepper Motor Force and Frequency

Measurements have shown that the T-26 motor can deliver 63 N of force at a pressure of 0.65 MPa. This force is approximately one order of magnitude higher than the minimum required force to pierce the breast skin with a sharp 14G needle (order of 10 N). The maximum stepping frequency in an MRI environment is limited to 8 Hz when maximum force is needed, corresponding to a motion speed of 2 mm/s, or 2°/s for rotational joints, but it can be reliably increased to 20 Hz for unloaded movements.

The T-26, and its curved counterpart, the C-30, can actuate all joints of the robot without chance of missing steps, provided that the operating pressure and stepping frequency are appropriate for handling the specific robot loads. From a known initial position full knowledge of the joint state vector during normal operation can be guaranteed by feed-forward control.

### Stormram 4 Accuracy

Measurements have shown that the robot achieves an accuracy of 0.73 ± 0.47 mm in free air, but the accuracy and precision in *X*-direction are not as good as in *Z*-direction. It was found that movement in the *Y*–*Z* plane is not precisely perpendicular to the *X* axis, causing measurable horizontal needle displacements when the needle is tilted up or down. This can be attributed to deficits in structural stiffness of the kinematic design and, combined with clearances in both linear joints, these result in measurable parasitic motions. When these factors are taken into account the horizontal accuracy could be improved to 0.2 mm. A better solution would be to improve the mechanical design by adding structural strength and reducing clearances in the different joints and links.

In MRI, the targeting error was found to be 1.30 ± 0.61 mm (range 0.44 to 2.85 mm). This error is larger than the accuracy in free air and can be mainly attributed to needle–tissue interactions which result in deflections of the needle. Other error sources are in imaging and registration: the used 3D bSSFP scanning sequence has an acquisition resolution of 1.5 × 1.5 × 2.0 mm and therefore relies on sub-pixel reconstruction accuracy for segmentation of both the fiducials and the needle shape. In a clinical setting the different field strengths and/or scanning sequences involved may result in different shapes of the needle artifacts, potentially resulting in higher or lower targeting errors. Lastly, a weak correlation between *Z*-position and *X*-error can be observed with correlation coefficient 0.5, which is in accordance with measurements in free air.

### Procedure Time

The mean procedure time per site, involving robot manipulation and performing the confirmation scan, was measured to be 6:38 min. This is equal to the total time of a 3D bSSFP scan, including pre-scan calibration and image reconstruction. An MRI scanner with a stronger magnetic field (e.g., 3 T) would allow quicker scans with the same SNR and resolution, potentially reducing the procedure time.

The robot needs less than 1:30 min to move the needle from one target site to another. This is mainly attributed to the needle insertion and retraction speed of 2 mm/s over a distance of up to 80 mm. When faster operation is desired it could be achieved by combining two stepper motors in a single linear joint with different step sizes, to allow both large and small steps to be made at the same stepping frequency of 8 Hz under load.

### Future Developments

Additional developments are needed before clinical trials can be considered. A breast fixation system integrated in a breast RF coil needs to be developed to immobilize a patient’s breast. The robot is to be equipped with a biopsy gun in order to take tissue samples. The structural stiffness should be improved to consistently maintain high accuracy. Safety mechanisms and procedures need to be developed for sterilization and also to cope with any possible system failure. When all these elements are incorporated, the robotic system has good potential for *in vivo* clinical use.

## Electronic supplementary material

Below is the link to the electronic supplementary material.

Supplementary material 1 (PDF 37 kb)

## References

[1] Abdelaziz, M. E. M. K., V. Groenhuis, J. Veltman, F. Siepel, and S. Stramigioli. Controlling the Stormram 2: an MRI-compatible robotic system for breast biopsy. In: IEEE International Conference on Robotics and Automation, pp. 1746–1753, 2017.

[2] Bomers JGR, Bosboom DGH, Tigelaar GH, Sabisch J, Fütterer JJ, Yakar D (2017). Feasibility of a 2nd generation MR-compatible manipulator for transrectal prostate biopsy guidance. Eur. Radiol..

[3] Chevrier MC, David J, El Khoury M, Lalonde L, Labelle M, Trop I (2016). Breast biopsies under magnetic resonance imaging guidance: challenges of an essential but imperfect technique. Curr. Probl. Diagn. Radiol..

[4] Franco E, Brujic D, Rea M, Gedroyc WM, Ristic M (2016). Needle-guiding robot for laser ablation of liver tumors under MRI guidance. IEEE/ASME Trans. Mechatron..

[5] Groenhuis V, Stramigioli S (2016). Laser-cutting pneumatics. IEEE/ASME Trans. Mechatron..

[6] Groenhuis, V., J. Veltman, F. J. Siepel, and S. Stramigioli. Design and characterization of Stormram 4: an MRI-compatible robotic system for breast biopsy. In: IEEE/RSJ International Conference on Intelligent Robots and Systems, pp. 928–933, 2017.

[7] Groenhuis, V., J. Veltman, and S. Stramigioli. Stormram 2: a MRI-compatible pneumatic robotic system for breast biopsy. In: Proceedings of the Hamlyn Symposium on Medical Robotics, pp. 52–53, 2016.

[8] Groenhuis V, Veltman J, Siepel FJ, Stramigioli S (2017). Stormram 3: a magnetic resonance imaging-compatible robotic system for breast biopsy. IEEE Robot. Autom. Mag..

[9] Lehman CD, DePeri ER, Peacock S, McDonough MD, DeMartini WB, Shook J (2005). Clinical experience with MRI-guided vacuum-assisted breast biopsy. Am. J. Roentgenol..

[10] Peters NHGM, Borel Rinkes IHM, Zuithoff NPA, Mali WPTM, Moons KGM, Peeters PHM (2008). Meta-analysis of MR imaging in the diagnosis of breast lesions. Radiology.

[11] Stoianovici D, Patriciu A, Petrisor D, Mazilu D, Kavoussi L (2007). A new type of motor: pneumatic step motor. IEEE/ASME Trans. Mechatron..

[12] Stoianovici D, Kim C, Srimathveeravalli G, Sebrecht P, Petrisor D, Coleman J, Solomon SB, Hricak H (2014). MRI-safe robot for endorectal prostate biopsy. IEEE/ASME Trans. Mechatron..

[13] Stoianovici D, Kim C, Petrisor D, Jun C, Lim S, Ball MW, Allaf ME (2017). MR safe robot, FDA clearance, safety and feasibility of prostate biopsy clinical trial. IEEE/ASME Trans. Mechatron..

[14] Torre LA, Bray F, Siegel RL, Ferlay J, Lortet-Tieulent J, Jemal A (2015). Global cancer statistics, 2012. CA Cancer J. Clin..

[15] Veltman J, Boetes C, Wobbes T, Blickman JG, Barentsz JO (2005). Magnetic resonance-guided biopsies and localizations of the breast: initial experiences using an open breast coil and compatible intervention device. Investig. Radiol..

