# Supplementary material for: Stormram 4: An MR Safe Robotic System for Breast Biopsy
Source: Ann Biomed Eng. 2018 May 21;46(10):1686–96. doi: 10.1007/s10439-018-2051-5 (PMC6153978; doi:10.1007/s10439-018-2051-5)
Supplement: Supplementary file 1 — Supplementary material 1 (PDF 37 kb) [file 10439_2018_2051_MOESM1_ESM.pdf]

Supplementary table with MRI experimental data

nr	site L	site P	site S	manual segmentation	insertion depth	needle diameter	azimuth	elevation
1	-19.9	-3.58	-1.32		14.04	4.44	1.08	-0.2
2	30.74	-15	23.97		24.86	7.07	1.41	-4.08
3	45.93	3.79	16.55	yes	21.94		0.57	-14.55
4	28.34	-5.71	19.45		21.49	4.65	1.83	-4.16
5	1.17	-13.09	-1.53		8.29	4.74	3.29	1.57
6	-11.76	-17.82	6.1		9.43	5.57	0.84	-1.24
7	-21.76	-15.05	13.75		15.33	5.47	-2.42	-0.13
8	37.43	2.85	27.4		35.95	4.41	0.74	-6.41
9	14.58	-4.62	-10.3	yes	11.23		0.69	-5.93
10	-27.84	-22.69	12.1	yes	11.8		-1.1	0.34
11	41.42	14.36	19.4		16.99	5.5	-0.12	-10.09
12	-20.21	1.05	13.29		18.71	5.71	1.26	-4.06
13	7.99	-13.13	-6.63	yes	10.25		1.96	-2.46
14	6.46	19.92	26.32		43.42	5.28	0.63	-11.45
15	16.78	-10.47	22.15	yes	12.42		-0.25	-3.94
16	41.87	-12.94	-2.48	yes	20.25		0.97	-7.47
17	25.92	-22.95	3.46		18.89	7.33	0.09	-5.03
18	-26.17	-30.92	7.53		25.67	6.77	0.53	-2.7
19	2.62	-27.86	8.07		28.76	5.89	0.99	-3.07
20	-8.97	-26.15	7.68		41.06	5.62	0.48	-3.58
21	-26.36	-20.78	11.47		31.36	5.89	1.29	-4.09
22	34.13	-28.51	14.39		33.97	5.5	0.94	-2.77
23	28.36	-35.14	25.25		29.03	7.27	-0.71	1.58
24	13.18	-21.64	-9.05		25.25	5.61	0.5	-5.89
25	-8.35	-27.28	2.18		26.98	6.1	0.93	-3.68
26	3.6	-40.45	13.23		26.55	6.22	1.5	0.9
27	5.58	-33.97	18.25		36.03	5.89	0.54	-1.25
28	34.61	-9.53	12.33		45.98	5.26	0.49	-10.07
29	-10.68	-13.12	-1.63		33.69	5.72	0.33	-9.25
30	9.61	-17.99	17.38		51.27	5.64	0.68	-6.91
mean	8.28	-14.95	10.29		25.03	5.73	0.67	-4.34
std	22.94	14.27	10.65		11.54	0.79	1	3.96
min	-27.84	-40.45	-10.3		8.29	4.41	-2.42	-14.55
max	45.93	19.92	27.4		51.27	7.33	3.29	1.58

normal error	3d error	X error	Z error	depth error	azimuth error	elevation error
1.44	3.57	0.16	1.43	3.27	0.72	5.5
1.59	3.49	-0.29	1.56	3.1	1.05	-2.58
2.04	2.06	0.48	1.92	-0.25	0.21	-2.55
1.26	2.95	0.48	-1.16	-2.67	1.47	0.34
0.66	0.87	0.51	-0.42	-0.56	2.93	-0.43
1.55	1.56	-0.22	-1.54	0.15	0.48	-0.24
0.6	1.01	-0.5	0.33	0.81	-2.78	1.37
1.53	1.54	1.43	-0.55	0.09	0.38	0.59
1.47	1.59	0.2	-1.45	0.61	0.33	0.07
0.97	2.03	0.93	0.29	1.78	-1.46	-0.16
2.51	3.04	-1.27	2.13	1.72	-0.48	1.91
1.25	2.08	1.2	0.34	1.67	0.9	3.44
0.43	1.2	0.43	-0.06	1.12	1.6	0.54
2.38	3.07	0.76	2.22	1.93	0.27	2.05
2.63	2.63	-1.67	2.03	-0.1	-0.61	-0.94
1.59	1.67	-1.22	-1.02	0.5	-0.49	2.03
1.11	2.74	-0.77	-0.8	2.5	-1.37	-0.03
0.63	2.14	-0.53	0.35	-2.05	-0.93	-0.2
1.22	1.32	-1.19	-0.26	0.5	-0.47	0.43
0.66	0.69	-0.62	-0.22	0.19	-0.99	-0.08
0.67	1.26	-0.52	0.42	1.06	-0.18	1.41
0.57	1.55	0.08	-0.57	1.44	-0.52	-0.27
2.2	2.23	-2.11	-0.63	0.36	-2.17	1.58
0.82	0.98	-0.81	0.17	0.52	-0.96	0.11
1.07	1.47	-0.78	-0.72	1.02	-0.54	-0.18
1.05	1.55	-1	-0.33	1.14	0.04	-0.1
1.09	1.1	-1	-0.41	-0.21	-0.92	-0.25
1.29	2.18	-1.16	-0.56	1.76	-0.97	-0.07
0.9	0.97	-0.79	0.43	0.35	-1.13	0.25
1.44	1.44	-1.17	-0.84	0.11	-0.78	-0.41
1.29	1.87	-0.25	0.44	0.73	-0.25	0.44
0.59	0.8	1.16	1.56	1.27	1.16	1.56
0.43	0.69	-2.78	-2.58	2.67	-2.78	-2.58
2.63	3.57	2.93	5.5	3.27	2.93	5.5
